# 

**Published:** 1895-10-05

**Authors:** 


CONTENTS.

**The Hospital: A Medical Journal** - 1

**Louis Pasteur** - 2

**On Modern Progress in Ophthalmic Medicine and Surgery.** By R. Brudenell Carter, F.R.C.S. - 3

**Medical Progress and Hospital Clinics—**

The Treatment of Patients after Abdominal Operations - 7

Progress in Medicine.—Diseases of the Stomach - 8

Progress in Surgery.—Brain Surgery - 10

Surgery of the Nerves - 11

Progress in Laryngology.—Pharynx - 12

Therapeutic Notes.—Argonine and Argentamine - 13

New Appliances and Things Medical - 14

**Social Problems.**—The Criminal Type - 4

**Annotations.**—The Classification of Paupers—Sunstroke—The Death of M. Pasteur - 5

**Notes and News** - 6

**The Institutional Workshop—**

Hospital Expenditure.—The Commissariat.—I. - 15

Practical Departments - 16

Hospital Festivals, Meetings, &c. - 17

Construction Notes - 17

**The Editor's Letter Box** - 18

**Scraps and Gleanings** - 18

**The Book World of Medicine and Science** - 19

EXTRA SUPPLEMENT.—THE NURSING MIRROR.

**News from the Nursing World.**—Nurses' Quarters and Recreation—Hospital for Children and Women—Success and Failure—Nurses at Lambeth—The Probationers' Prizes—Surgical Home for Boys—Local Criticism of Coventry Guardians—A New Society—Twelve to Sixteen—The Indian Nursing Service—Our Needlework Competition—Over-managed - i
**Elementary Physiology for Nurses.** By C. F. Marshall, late Surgical Registrar, Hospital for Sick Children, Great Ormond Street - iii
**Nursing in Ireland** - iii
**Neuralgia and its Treatment.**—I. - iv
**Dress and Uniforms.**—St. Mary's Hospital - v
**Novelties for Nurses—Nursing in Rome.**—III. - vi
**French Schools for Trained Nurses: Their Origin and Organisation** - vii
**Holidays and Health.**—Ireland—**Where to Go** - viii
**Nurses in the Provinces.**—Training - ix
**The Ockley Scheme.**—The Name of Nurse - x
**One Aspect of Tommy Atkins—Notes from Melbourne** - vi
**Everybody's Opinion** - xii

